# Socioeconomic Status, Social Support, and Quality of Life Among Black Adults

**DOI:** 10.1007/s40615-024-02204-9

**Published:** 2024-10-30

**Authors:** Elijah R. Murphy, Guillermo M. Wippold, Zion R. Crichlow

**Affiliations:** 1 Department of Psychology, University of Houston, Houston, USA; 2 Department of Psychology, University of South Carolina, Columbia, USA

**Keywords:** Socioeconomic status, Social support, Quality of life

## Abstract

**Objectives:**

Socioeconomic status has a pervasive influence on one’s health and quality of life. Social support is known as a factor that can minimize the risk of maladaptive health outcomes while promoting greater quality of life. The purpose of this study was to examine the direct and indirect relationships between perceived socioeconomic status, social support, physical quality of life, and psychological quality of life among Black adults.

**Methods:**

Participants were 304 (118 men, 186 women) adults who identified as African American or Black. Ages ranged from 18 to 75 (Mage = 37.62 years, SD = 12.39 years) and completed a battery of self-report questionnaires on perceived socioeconomic status, social support, physical quality of life, and psychological quality of life.

**Results:**

Structural equation modeling analyses demonstrated that perceived socioeconomic status is associated with greater social support, physical quality of life, and psychological quality of life. Perceived socioeconomic status was also associated with higher physical quality of life and psychological quality of life indirectly through more social support.

**Conclusions:**

These findings provide evidence that perceived socioeconomic status is directly associated with greater quality of life in Black adults. Social support acts as a partial mediator in the relationship between socioeconomic status and quality of life. This research brings to attention the influence that socioeconomic status and social support have on the quality of life of Black adults. These factors are of particular importance when considering social determinants of health in Black communities.

## Health-Related Quality of Life

Health-related quality of life (HRQoL) is a multidimensional indicator of health and well-being that consists of an individual’s subjective perception of their psychological and physical functioning [1–3]. HRQoL has gained attention because low HRQoL is a strong predictor of premature mortality [4]. Given its association with premature mortality, HRQoL has been a national public health priority since the early 2000s with the first iteration of the Department of Health and Human Services’ Healthy People Campaign — a campaign that set national objectives to improve the health of Americans. Despite decades of efforts to promote HRQoL among Americans, stark disparities exist — Black Americans report significantly lower HRQoL than White Americans [5, 6]. This disparity is reflected in life expectancy estimates. Black Americans have among the lowest life expectancy in the United States [7]. Black Americans have a life expectancy of 70.8 years, which is less than that of Asian Americans (83.5 years), non-Hispanic White Americans (76.4 years), and Hispanic Americans (77.1 years) and only greater than that of non-Hispanic American Indian or Alaska Natives (65.2 years) [7]. Community-informed efforts are needed to promote HRQoL and increase life expectancy among Black Americans. There is strong support for such efforts that involve Black Americans in the development and have been successfully implemented in barbershops, beauty salons, primary health clinics, and predominately Black churches [8–12].

## Socioeconomic Status and HRQoL

Socioeconomic status (SES) is an economic and sociological composite indicator that encompasses income, education, occupation, subjective social status, and social class. It is well-established that SES has a significant influence on health. People with more socioeconomic advantages tend to have better health outcomes [13–18]. For example, it is known that SES is positively correlated with HRQoL [19, 20]. Explanations for the association between SES, health, and HRQoL have been explored and identified. Indicators of SES, such as access to healthcare services and environmental risk exposure, are known to impact health and health outcomes [21–24]. Because of its strong relationship with health, SES has been considered an important factor in understanding health disparities in the United States. The relationship between SES and health has been of particular attention among Black Americans given that due to systemic discrimination, Black Americans are more likely to live in communities with fewer access to health resources [25, 26] and more environmental risk. Thus, in addition to policies and societal changes to improve the SES of Black Americans, there is a need to understand mechanisms to mitigate the impact of SES on HRQoL among Black Americans. Such mechanisms can be leveraged in health promotion efforts.

## Social Support as a Buffer Against the Adverse Effects of SES on Health Among Black Americans

Despite the well-documented impact of systemic inequities on the health and well-being of African Americans, a noteworthy quote in an article by Chae and colleagues 2021 attributed to James Jackson asks, “given the structural impediments that they [Black Americans] face, why do Black people do so well?” This quote calls for a shift in social research from a deficit-based approach to a strengths-based approach [27]. Following the need for this shift, research has sought to understand the health benefits of social support among Black Americans [28–33]. Social support (i.e., one’s perception and experience with being cared for and being socially integrated) has been established as a significant contributor to health and well-being [34–39]. Research has found that social support can be influential in the relationship between SES and health concerns across the lifespan [40]. For example, one study found that SES is positively associated with social capital and that social capital mediated the relationship between SES and quality of life [19]. Another study found that social support mitigated the impact of stress on HRQoL among older African Americans [41, 42].

Social support may be a culturally unique health-promoting mechanism among Black Americans. Social support has been seen as a factor that may buffer existing health disparities. It was found that social support in the form of asking a friend or family member for help protected against the negative effects of financial concerns on psychological distress in Black older adults, but not White older adults [43]. In a comparison between Black Americans and White Americans, Black Americans had decreased risk of depression than non-Hispanic White Americans while social support in the form of being close to family members was a heavily influencing factor in depression odds [44]. In addition, more social support is associated with lower blood pressure and other cardiovascular risk factors [45, 46]. Even in the case of a pervasive stressor for Black adults, discrimination and social support were protective factors in the relationship between discrimination and psychological distress [47]. Given social support’s impact and prevalence on outcomes in Black adults, it can be hypothesized that social support would have adaptive impacts on one’s perceived quality of life.

## Current Study

**Fig. 1:**
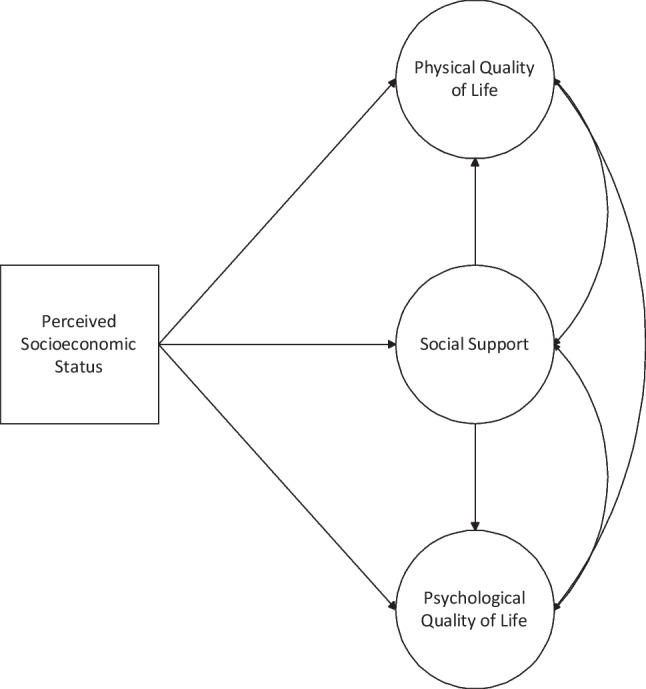
Conceptual framework

Despite the documented relationships between HRQoL, SES, and social support, no known study has investigated these relationships among Black Americans. Based on the Buffering Hypothesis [48], the hypotheses of the present study were that among a sample of Black Americans:

1. SES will have a significant and positive relationship with physical and psychological quality of life.
2. SES will have a significant and positive relationship with social support.
3. Social support will have a significant and positive relationship with physical and psychological quality of life.
4. SES will have an indirect association with physical and psychological quality of life through greater social support. The conceptual framework for this research can be found in Fig. 1.

## Method

### Participants

Participants were eligible for the present study if they (1) were 18 years of age or older, (2) identified as Black American or African American, (3) had access to the internet, (4) resided in the United States, and (5) could read in English. A total of 304 participants met the eligibility criteria and were included in the present study. See Table 1 for demographic information.

**Table 1:** Participant characteristics (*N* = 304)

Variable	Mean (*SD*)/*n* (%)
**Gender**^**a**^	
Women	186 (61.2%)
Men	118 (38.8%)
**Race/ethnicity**^**a**^	
Black/African American	304 (100%)
Not Hispanic/Latino	304 (100%)
**Age**^**a**^	37.62 (12.39)
**Occupational status**^**a**^	
Employed	225 (74%)
Unemployed	79 (26%)
**Education**^**a**^	
High school or GED	33 (10.9%)
Some college	74 (24.3%)
Trade/technical school	9 (3%)
2 year college	36 (11.8%)
4 year college	111 (36.5%)
Professional/graduate school	41 (13.5%)
**Annual income**^**a**^	
Less than $10,000	49 (16.1%)
$10,000–$19,999	35 (11.5%)
$20,000–$29,999	36 (11.8%)
$30,000–$39,999	43 (14.1%)
$40,000–$49,999	36 (11.8%)
$50,000 to $59,999	25 (8.2%)
$60,000 to $69,999	24 (7.9%)
$70,000 to $79,999	14 (4.6%)
$80,000–$89,999	13 (4.3%)
$90,000–$99,999	8 (2.6%)
More than $100,000	21 (6.9%)
**Perceived socioeconomic status**^**a**^	
10–high	9 (3%)
9	18 (5.9%)
8	51 (16.8%)
7	47 (15.5%)
6	61 (20.1%)
5	54 (17.8%)
4	47 (15.5%)
3	11 (3.6%)
2	1 (0.3%)
1–low	5 (1.6%)

^a^Demographics questionnaire

### Measures

#### Demographic Questionnaire

A researcher-created demographic questionnaire was developed and administered. This questionnaire assessed the following: (1) gender, (2) age, (3) race/ethnicity, (4) occupational status, (5) highest level of education attained, and (6) annual income.

#### ***The Medical Outcomes Study (MOS) Social Support Survey ***[49]

The MOS Social Support Survey is a 19-item survey that assesses perceived social support. There are four subscales: (1) emotional/information support, (2) tangible support, (3) affectionate support, and (4) positive social interaction. The total score is calculated by taking the average of all subscales. The subscale totals for tangible support, affectionate support, and positive social interaction were used for the present study. Higher scores indicate higher levels of social support. The MOS has demonstrated acceptable reliability among samples including Black Americans [31, 32].

#### The World Health Organization Quality of Life–Brief Form (WHOQOL-BREF)

The WHOQOL-BREF measures an individual’s perceptions of their quality of life across several health-related domains (physical, psychological, social, and environmental). The present study used 14 items from the WHOQOL-BREF that assessed the physical and psychological subscales of this measure. Higher scores indicate higher levels of HRQoL. The WHOQOL-BREF has demonstrated acceptable reliability among samples including Black Americans in several empirical studies [9, 10, 12].

#### ***The MacArthur Scale of Subjective Social Status ***[50]

The MacArthur Scale of Subjective Social Status (MSSSS) is a self-report, ladder-shaped scale used to measure perceived social status. Individuals who complete the MSSSS are asked to select the rung on the ladder-shaped scale that corresponds with their status. Higher rungs have higher scale scores and indicate higher perceived SES. The MSSSS has demonstrated acceptable reliability among samples including Black Americans in previous research [51, 52].

### Procedure

The present study received Institutional Review Board approval at a large public university in the Southeastern United States. A convenience sample of participants was recruited through MTurk — an online platform often used by social scientists. Users of MTurk have the opportunity to complete “tasks” (e.g., a survey about health) for compensation. The research team paid an additional fee to limit the availability of the survey to users who (1) identified as Black American or African American and (2) had an IP address in the United States. All users of MTurk must be 18 years of age or older. The survey title and compensation rate (i.e., $2.50) appeared on the dashboard of all eligible users of MTurk. Users could click on the survey and read a brief description of the procedures. Individuals who were interested in completing the survey were then directed to a consent form. This consent form indicated that the survey was confidential and asked participants to omit their names or other identifying information on any of the survey materials. The estimated completion time of the survey was 15 min.

### Analytic Procedure

**Fig. 2:**
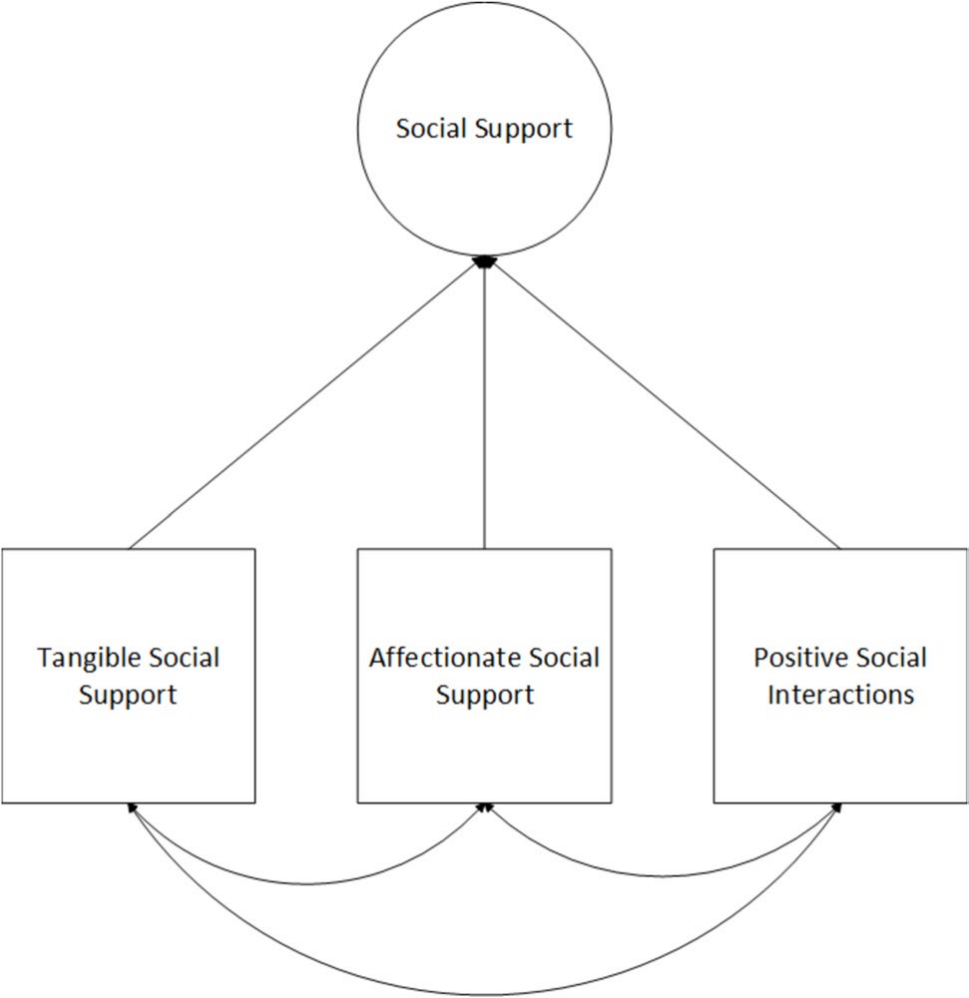
Social support as a latent construct

Structural equation modeling (SEM) with Mplus version 8 [53] was used to estimate the observed and latent relationships between socioeconomic status, social support, in addition to physical and psychological quality of life. Robust maximum likelihood estimation was used to account for the minimal missing data. Parcels were constructed for both psychological and physical quality of life to increase reliability in our latent constructs. Three parcels were created for each quality-of-life outcome by randomly assigning their individual items into parcels that were then used as manifest indicators of psychological and physical quality of life, respectively. Social support was identified as a latent construct by utilizing the three subscale totals of the MOS as observed variables: tangible social support, affectionate social support, and positive social interactions (Fig. 2). Perceived socioeconomic status remained as one observed indicator.

Covariates of age and gender were approximated and controlled for in subsequent analyses. First, direct relationships between perceived socioeconomic status, social support, physical quality of life, and psychological quality of life were estimated. The indirect relationships between perceived socioeconomic status on quality of life through social support were estimated using the MODEL INDIRECT command in Mplus based on 95% confidence intervals. Model fit for the SEM analyses was evaluated using the recognized model fit indices: the root mean square error (RMSEA), the Comparative Fit Index (CFI), the Tucker-Lewis Index (TLI), chi-squared (*χ*^2^), and standardized root mean squared residual (SRMR). If the CFI and TLI are higher than 0.90 [54] and the RMSEA value is lower than 0.05 or between 0.05 and 0.08 [55], the model is an acceptable fit for the data.

## Results

### Preliminary Analyses

Descriptive statistics for all study variables can be found in Table 2. Perceived socioeconomic status, quality of life, and social support were positively correlated as anticipated. Specific domains of social support (affectionate and tangible), as well as domains of quality of life (physical and psychological), were positively correlated with each other.

**Table 2:** Descriptive statistics and bivariate correlations among key constructs

	**1**	**2**	**3**	**4**	**5**	**6**
1. Tangible social support	-					
2. Affectionate social support	0.90^**^	-				
3. Positive social interactions	0.89^**^	0.90^**^	-			
4. Physical quality of life	0.32^**^	0.29^**^	0.30^**^	-		
5. Psychological quality of life	0.44^**^	0.44^**^	0.41^**^	0.65^**^	-	
6. Perceived socioeconomic status	0.37^**^	0.37^**^	0.33^**^	0.23^**^	0.39^**^	-
Means	14.69	10.71	10.71	15.60	14.39	4.9
Standard deviations	4.15	3.16	3.29	3.04	3.36	1.9

**p < 0.001

### Age and Gender as Covariates

Age and gender identity were accounted for in all analyses. Regarding age, there were small, positive relationships between age and both physical (*β* = 0.19, 95% CI = 0.16:0.40) and psychological HRQoL (*β* = 0.17, 95% CI = 0.16:0.40). This relationship suggests that as age increased, HRQoL increased, meaning that older participants reported higher HRQoL. Regarding gender, gender identity had small, negative associations with both physical (*β* =  − 0.23, 95% CI =  − 0.33: − 0.13) and psychological HRQoL (*β* =  − 0.20, 95% CI =  − 0.30: − 0.10). In this sample, men were coded as “1” and women were coded as “2.” This relationship suggests that participants who identified as men reported more HRQoL than did participants who identified as women. Neither age nor gender was significantly associated with social support.

### Relationships Between SES, Social Support, and HRQoL

SEM analyses were conducted to observe the relationships between SES, social support, physical HRQoL, and psychological HRQoL (Table 3). The model fit of the SEM was excellent (CFI = 0.963, TLI = 0.946, ${x}^{2}$ =102.89 (df = 44), RMSEA = 0.066, SRMR = 0.059). Higher SES had a small association with greater physical HRQoL (*β* = 0.28, 95% CI = 0.16:0.40, *R*^2^ = 0.30) and a medium association with more psychological HRQoL (*β* = 0.32, 95% CI = 0.22:0.43, *R*^2^ = 0.41). Higher SES also had a medium association with more social support (*β* = 0.37, 95% CI = 0.26:0.48, *R*^2^ = 0.14).

**Table 3:** Direct relationships between socioeconomic status and quality of life

Outcome	*b*	*Se*	*p*	*β*	95% CI	*R*^2^
Physical quality of life	0.12	0.03	< 0.001	0.28	0.16:0.40	0.30
Psychological quality of life	0.15	0.03	< 0.001	0.32	0.22:0.43	0.41
Social support	0.78	0.01	< 0.001	0.37	0.26:0.48	0.14

*b* represents the unstandardized beta coefficient; *β* represents the completely standardized beta coefficient. *CI* represents 95% standardized confidence intervals. *SE* represents standard error

### Indirect Relationships Between SES and HRQoL Through Social Support

SEM analyses revealed indirect relationships between SES and HRQoL through social support. As seen in Table 4, higher social support had a medium association with both greater physical HRQoL (*β* = 0.29, 95% CI = 0.17:0.41) and psychological HRQoL (*β* = 0.39, 95% CI = 0.27:0.51). Through greater social support, higher SES had a small indirect association with both higher physical quality of life HRQoL (ab = 0.11, 95% C.I = 0.05:0.16). Similarly, greater SES had a small indirect association with higher psychological HRQoL through greater social support (ab = 0.14, 95% C.I = 0.08:0.20).

**Table 4:** Relationships between social support and quality of life

Outcome	*b*	*Se*	*p*	*β*	95% CI	*R*^2^
Physical quality of life	0.06	0.01	< 0.001	0.29	0.17:0.41	0.30
Psychological quality of life	0.09	0.01	< 0.001	0.39	0.27:0.51	0.41

b represents the unstandardized beta coefficient; *β* represents the completely standardized beta coefficient. *CI* represents 95% standardized confidence intervals. *Se* represents standard error

## Discussion

Surveillance data indicates that Black Americans experience disproportionately high rates of low health-related quality of life [56] — an indicator of health strongly associated with premature mortality [57]. Efforts are needed to promote this indicator of health among Black Americans, though such efforts must be informed by formative research conducted among these individuals [58]. That is because there are unique influences of culture on health. Research has highlighted the unique role of social support in mitigating adverse health experienced by Black Americans [20]. Thus, the purpose of the present study was to investigate the impact of socioeconomic status and social support on health-related quality of life among Black Americans.

The findings from the present study found that socioeconomic status and health-related quality of life were positively associated, suggesting that perceived high socioeconomic status was associated with better health-related quality of life. This suggests that the inverse is also true: perceived low socioeconomic status is associated with worse health-related quality of life. This present research also suggests that although perceived low socioeconomic status had a deleterious effect on both physical and psychological health-related quality of life among the participants, the association between perceived low socioeconomic status and health-related quality of life was mediated by social support. Meaning that perceived high SES is associated with better HRQOL when social support is higher, and that perceived low SES is associated with lower HRQOL when social support is lower. These results confirm previous findings that have reported a significant relationship between socioeconomic status and health-related quality of life [41, 42]. With that being the case, no known study has examined perceived socioeconomic status as a predictor of health-related quality of life among a sample consisting of only Black and/or African American participants. The present study builds on this novel finding by further examining the partial mediating role of social support in the relationship between perceived socioeconomic status and health-related quality of life among Black and/or African American individuals. This finding aligns with previous studies among samples consisting solely of Black and/or African American individuals that found that a sense of a community (i.e., a concept similar to social support, yet distinct) mitigated the impact of adverse health behaviors on health-related quality of life [11]. Additionally, this finding is similar to previous findings that found that loneliness (i.e., a subjective experience of little social support) was negatively associated with health-related quality of life among older Black adults [41, 42]. In addition to being a sample consisting solely of Black adults, it is noteworthy that the findings from the present study are among a young sample (mean age = 37.62) of Black adults.

The findings from the present study have important implications for efforts to promote health-related quality of life among Black individuals. There are well-documented difficulties in recruiting, retaining, and producing meaningful health-related change among Black Americans in health efforts [59, 60]. It has been suggested that past efforts have been limited in impact because they have been too narrow in scope (i.e., focusing on one indicator of health) [61]. Qualitative research among Black Americans indicates that these individuals have a holistic conceptualization of health — one that aligns well with the concept of health-related quality of life [62, 63]. Thus, efforts to promote health-related quality of life among Black Americans may hold promise to promote health equity. Additionally, past research has found that Black Americans prefer to rely on informal sources (e.g., peers, family members) of support to promote health [9, 10, 64]. Previous successful efforts have leveraged social support to improve health among Black adults, though the findings from this study support the use of social support to improve health-related quality of life. An effort to promote health-related quality of life among Black Americans by intentionally addressing social support may align the effort with the values and preferences of these individuals. Such alignment is known to promote successful uptake and meaningful health-related changes among Black Americans [12].

The present study also has notable policy implications. The findings underscore the importance of addressing socioeconomic status as a social determinant of health. There is a need for policies to address disparities in socioeconomic status among Black and African Americans. Such policies should target educational opportunities, healthcare services, and employment opportunities — critical determinants of socioeconomic status [65]. In addition to improving disparities in socioeconomic status, such policies could have a ripple effect and secondarily improve social support and health-related quality of life. It is likely that policy efforts would have a more widespread and sustainable impact on health inequalities beyond individual and community-level health promotion efforts.

The findings of the present study and the conclusions drawn from these findings should be viewed considering the limitations and strengths of the present study. The primary limitations of the present study include the following: (1) the use of an internet-based sample, (2) the cross-sectional design of the study, and (3) the use of self-report measures. It should be noted that participants were recruited from Amazon Mechanical Turk — a web-based platform commonly used for social science research [66]. The use of this platform may limit the generalizability of the findings given that African American/Black users of mTurk do not mirror the African American/Black population of the United States [66]. The sample of the present study had a higher educational attainment than the average sample of African American/Black individuals [67]. Despite this limitation, the use of mTurk did yield an added benefit — the mean sample age was only 37.8 years old. Younger African American and Black individuals are often absent from health promotion research [60] and there is an increasing need for preventive health efforts to curb future adverse health conditions. In addition to this limitation, the present study was cross-sectional in nature, which limits inferences of causality. Additionally, the present study used self-report measures, which are known to increase bias (Van [68]). A final limitation of this study is that this study did not account for certain potential confounding variables (e.g., employment, education, and access to care), although many of these variables are subsumed under the broader construct of SES. With that being the case, future studies should aim to parse out the unique significance of these variables to provide a more nuanced understanding of their impact on HRQoL.

Despite these limitations, the strengths of the present study are noteworthy. First, the sample consisted solely of individuals who identify as Black and/or African Americans. According to the National Academy of Science, Engineering, and Medicine, there is an underrepresentation of Black/African American individuals in health promotion research and that such underrepresentation has stymied efforts to promote health equity [60]. Thus, the present study contributes to a much-needed literature base. Additionally, the analytic approach used in the present study (i.e., structural equation modeling) is robust and increases the confidence in the findings by limiting the influence of measurement error. Finally, the investigation of a mediating factor — social support — presents a viable solution to mitigate the impact of socioeconomic status on health-related quality of life among Black Americans. Future efforts should investigate strategies to bolster social support (e.g., support from family, friends, and community) in order to promote health among Black and/or African Americans. Such sources are cost-effective and sustainable. Furthermore, it is important to note that this study used an aggregate of social support. Future research may benefit from understanding which type of support (e.g., tangible, affectionate, positive social interactions) and from which source (e.g., family, friends, and community) yield the strongest impacts. In conclusion, there is a need to promote health-related quality of life among Black Americans — a group that reports low health-related quality of life, which is an indicator of health strongly associated with premature mortality. Such efforts should be based on formative research and be contextually/culturally salient. The present study found that social support mitigated the impact of socioeconomic status on physical and psychological health-related quality of life. Health promotion and policy efforts that target socioeconomic status and social support may play an important role in promoting health equity among Black Americans.
